# Cystic Fibrosis-Related Diabetes and Subclinical Hypothyroidism in Pregnancy

**DOI:** 10.7759/cureus.8895

**Published:** 2020-06-28

**Authors:** Ioannis Kakoulidis, Ioannis Ilias, Anastasia Linardi, Evangelia Venaki, Eftychia Koukkou

**Affiliations:** 1 Department of Endocrinology, Diabetes and Metabolism, Elena Venizelou General and Maternity Hospital, Athens, GRC

**Keywords:** cystic fibrosis, cfrd, diabetes, subclinical hypothyroidism, pregnancy

## Abstract

Pregnancy in women with cystic fibrosis-related diabetes (CFRD) is rare and requires intensive monitoring and individualized treatment due to the pathophysiologic parameters of the disease in relation to insulin therapy and special nutritional needs. We present the case of a 33-year-old primigravida woman with CFRD (ΔF508 homozygote, with mild pulmonary involvement) on insulin therapy and treatment for exocrine pancreatic insufficiency, who developed subclinical hypothyroidism during gestation. Due to the complexity of the disease, major clinical challenges were glycemic variance, hypoglycemic episodes, and difficulty in weight gaining. In addition, the presence of malabsorption in the intestinal mucosa was an important aspect of difficulty in the treatment of subclinical hypothyroidism. Thus, the flexible approach in the timing of basal insulin administration, combined with the individualized medical nutrition therapy, and along with the progressive increase in levothyroxine dosage, all were proven to be key components in the effective management of our patient.

## Introduction

Cystic fibrosis-related diabetes (CFRD) is a separate clinical entity between diabetes type 1 and type 2, with simultaneous presence of insulin deficiency and insulin resistance, along with malabsorption in the intestinal mucosa. It is present in 20% of adolescents and 40%-50% in adults with cystic fibrosis (CF). CFRD is a co-morbidity factor to the already established pulmonary disease of CF with adverse effects on maternal survival (especially when forced expiratory volume in 1 second [FEV1] is less than 60%), short-term fetal outcomes, related to preterm delivery (10%-25% of cases), and increased rates of low birth weight (<2.5 kg). Pregnancy in women with CFRD is relatively rare, requiring intensive monitoring due to the disease’s unique pathophysiologic characteristics [1-7].

## Case presentation

**Figure 1:**
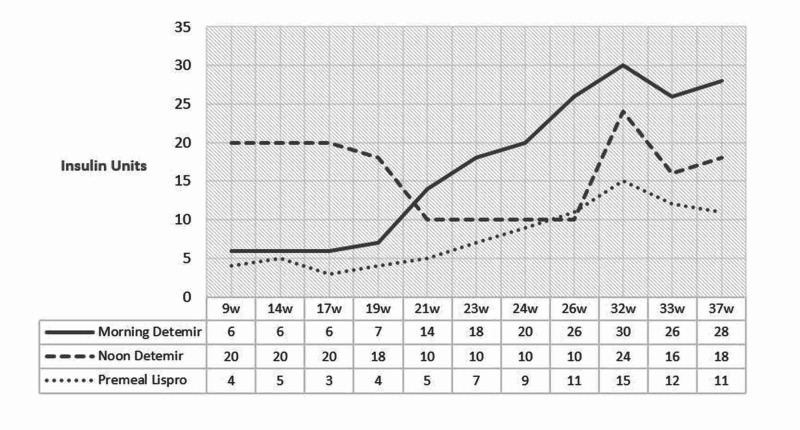
Mean insulin dosage variance in gestation

Α 33-year-old woman (47 kg, BMI 20.6 kg/m^2^), non-smoker, with regular menstrual cycle of 32 days and CF (diagnosed at age of seven years; homozygote ΔF508/ΔF508, with mild pulmonary involvement - FEV1 >60%) who developed CFRD at age 28 years (treated with insulin), presented during the first trimester of pregnancy (7^+2^ weeks primigravida, following intrauterine insemination, without having a previous genetic counseling) in the endocrine clinic for further monitoring. She was treated, based on the pre-pregnancy treatment regimen, with insulin glargine 16 units in the evening and insulin glulisine 8 to 10 units before meals, with increased distribution of carbohydrates and fats. The patient’s glycemic profile ranged between 70 and 80 mg/dl fasting and 90 and 140 mg/dl two hours postprandial (occasionally up to 200 mg/dl, HbA1c 5.9%). She was also receiving pancreatin enzymes [Creon 25,000 IU (300 mg) two caps/meal] and multivitamins. Based on the patient's history and the likelihood of presence of hypoglycemic episodes alternating with hyperglycemia, depending on the degree and rate of food absorption, the patient's treatment was modified in correspondence with the CFRD guidelines [1,3]. Basal insulin detemir of six units in the morning and 20 units in the evening was initiated, along with insulin lispro four to six units at one hour postprandial, if blood glucose levels were above 150-160 mg/dl. Self-monitoring of glycemia, with point of care devices, confirmed the presence of severe morning hypoglycemic episodes. A more individualized approach based on the nutritional needs of the patient combined with the maintenance of the feeding time plan (three main meals combined with intermediate smaller ones) initially improved and then resolved the hypoglycemic episodes. More basal insulin was gradually administered reaching 28 units of detemir in the morning and 18 units at evening, with insulin lispro of 10-12 units premeal. This plan significantly improved glycemic fluctuations/24 hours. Furthermore, the rate of weight change was also improved with a satisfactory total gain of 7 kg in pregnancy. Since this was a high-risk pregnancy, and there was a significant possibility of premature delivery, the treating obstetrician sought to administer, at the 32^nd^ week of gestation, 12+12 mg of betamethasone (24 hours apart). The patient was hospitalized for six days with intensive monitoring. A transient disturbance in the glycemic profile was noted, lasting on average 72 hours, with a corresponding increase in insulin needs (55% in her detemir and 114% in her lispro insulin dosage) (Figure 1).

Due to subclinical hypothyroidism of gestation, the patient was already on levothyroxine treatment (initial thyroid-stimulating hormone [TSH] 3.40 mIU/L with a starting dose of 50 mcg/day). An increase in TSH at 7.00 mIU/L was noted at the first visit, leading to an increase in levothyroxine dosage at 62 mcg/day. Nevertheless, despite treatment, levels of TSH remained high at 6.20 mIU/L with low levels of fT4 and fT3. A question was raised regarding the possible presence of malabsorption of levothyroxine in the intestinal mucosa. Eventually, a progressive increase in treatment at 100 mcg/day, for a short time, improved TSH levels. The patient was on 88 mcg/day before delivery (TSH 1.12 mIU/L).

At gestational age 38^+6^ weeks, with an uncomplicated fetal ultrasound, a cesarean section was performed. The healthy neonate weighted 3,070 g. Glycemic profile variability after delivery was satisfactory without hypoglycemic episodes. Levothyroxine was given at a lower dose (62 mcg/day), along with insulin treatment based on pre-pregnancy dosing.

## Discussion

Pregnancies of women with CF are managed as high risk (premature delivery often occurs in 9% to 35% of pregnancies with CF), especially when they are complicated by diabetes [6]. In these rare cases, the most difficult task for the health care provider is to balance the nutritional requirements of pregnancy in a woman with CFRD, and at the same time to maintain glycemic control [7]. In our case, the gradual switch in basal insulin administration with more units in the morning than in the evening improved the variance of glycemia in the 24-hour period, and resolved the hypoglycemic episodes. This was extremely important, especially during the betamethasone administration, since a significant increase in insulin dosage was mandatory to encounter with the disturbance in the glycemic profile. The diabetogenic potential of corticosteroids is known, and combined with placental insulin resistance in pregnancy, leads to a transient increase in blood glucose levels of pregnant women. In order to control the glycemic variance resulting from this effect, it is recommended to hospitalize patients especially when they are on insulin therapy [8,9]. The rate of weight change was also improved with a satisfactory total gain of 7 kg in pregnancy, considering that weight gain is often difficult among pregnant women with CFRD [3,4,7]. The recommendation for CF in pregnancy is to eat a high-fat and energy diet (120%-150% of the recommended energy intake for pregnant women without CF) in order to increase the maximum absorption rate as much as possible, aiming for a weight gain of 11 kg [7]. Additionally, pancreatin enzymes and multivitamins help to counter the variance in rhythm and degree of food absorption in the intestinal mucosa. Regarding the levothyroxine treatment and the possible presence of malabsorption of levothyroxine in the intestinal mucosa in women with CF, a progressive increase in dosage at 100 mcg/day, for a short time, improved TSH levels. The outcome of this pregnancy was uneventful, without complications in the perinatal period based on her mild pulmonary involvement and her satisfactory clinical status after insulin and levothyroxine treatment interventions.

## Conclusions

Pregnancy in women with CFRD is rare and requires intensive monitoring and individualized treatment due to the pathophysiologic parameters of the disease in relation to insulin therapy and special nutritional needs. We present the case of a 33-year-old woman in her first pregnancy with CFRD, on insulin therapy and treatment for exocrine pancreatic insufficiency, who developed subclinical hypothyroidism during gestation. Our patient presented clinical challenges regarding glycemic variance, hypoglycemic episodes, and difficulty in weight gain. In addition, the presence of malabsorption in the intestinal mucosa played an important role in the efficacy of treatment for subclinical hypothyroidism. Thus, the flexible approach in the timing of basal insulin administration, combined with the individualized medical nutrition therapy, and along with the progressive increase in levothyroxine dosage, all were proven to be key components in the effective management of our patient. Since there is a lack of similar cases in the literature, we strongly believe that sharing our experience could be helpful to physicians who manage pregnant women with CF or CFRD.
